# Caesarean Section for Placenta Previa: A Retrospective Cohort Study of Anaesthesia Techniques

**DOI:** 10.5152/TJAR.2023.22789

**Published:** 2023-02-01

**Authors:** Samina Ismail, Saima Rashid

**Affiliations:** Department of Anaesthesiology, Aga Khan University, Karachi, Pakistan

**Keywords:** Anaesthesia techniques, blood loss, blood transfusion, caesarean section, neonatal outcomes, placenta previa

## Abstract

**Objective:**

Placenta previa is associated with maternal and neonatal morbidity and mortality. This study aims to add to the limited literature from the developing world on the association of different anaesthetic techniques with blood loss, the need for blood transfusion, and maternal/neonatal outcomes among women undergoing caesarean section with placenta previa.

**Methods:**

This retrospective study was conducted at Aga University Hospital, Karachi, Pakistan. The patient population included parturients undergoing caesarean section for placenta previa from January 1, 2006, through December 31, 2019.

**Results:**

Out of 276 consecutive cases of placenta previa progressing to caesarean section during the study period, 36.24% were performed under regional anaesthesia and 63.76% under general anaesthesia. When compared to general anaesthesia, significantly less regional anaesthesia was used for emergency caesarean section (26% vs. 38.6%, *P* = .033) and for grade IV placenta previa (50% vs. 68.8%, *P* = .013). Blood loss was found to be significantly low with regional anaesthesia (*P* = .005) and posterior placenta (*P* = .042), while it was found to be high in grade IV placenta previa (*P* = .024). The odds of requiring blood transfusion were low in regional anaesthesia (odds ratio = 0.122; 95% CI = 0.041-0.36, *P* = .0005) and posterior placenta (odds ratio = 0.402; 95% CI = 0.201-0.804, *P* = .010), while they were high in grade IV placenta previa (odds ratio: 4.13; 95% CI = 0.90-19.80, *P* = .0681). The rate of neonatal deaths and intensive care admission was significantly lower in regional anaesthesia than in general anaesthesia (7% vs. 3% and 9% vs. 3%). The maternal mortality was zero; however, intensive care admission was less in regional anaesthesia compared to general anaesthesia (<1% vs. 4%).

**Conclusion:**

Our data demonstrated less blood loss, need for blood transfusion, and better maternal and neonatal outcomes with regional anaesthesia for caesarean section in women with placenta previa.

**Main Points**

- Placenta previa is associated with maternal and neonatal morbidity and mortality.
- Caesarean section is the recommended mode of delivery for pregnancies with placenta previa.
- Limited literature is available from the developing world on anaesthesia techniques and placenta previa.
- Technique of anaesthesia appeared as a predictive factor for blood loss and blood transfusion.
- Regional anaesthesia is associated with less blood loss and the need for transfusion.
- General anaesthesia is associated with poor maternal and neonatal outcomes.

## Introduction

According to the world region statistics, the estimated prevalence of placenta previa (PP) mounts to 5.2 per 1000 pregnancies, with the highest prevalence quoted among the Asian population (12.2 per 1000 pregnancies).^1-3^ Placenta previa is associated with maternal and neonatal morbidity and mortality.^1^ It is a major risk factor for life-threatening maternal haemorrhage, preterm deliveries, stillbirths, and maternal/neonatal intensive care admission.^3,4^ Placenta previa is characterized into major and minor by the location of the placental edge in relation to the internal cervical os. In major PP, the placenta overlaps the internal cervical os completely (grade IV) or partially (grade III). Minor PP (grade II) is also defined as low-lying or marginal PP where the placenta is located <20 mm from the internal cervical os.^5,6^

Caesarean section (CS) is identified as the only safe and recommended mode of delivery for pregnancies with PP.^1^ This makes the role of anaesthesiologists very important and entices debate about the best anaesthetic option to employ.^1,7^ Due to an anticipated increased risk of blood loss in these patients, general anaesthesia (GA) is preferred by some anaesthesiologists, while others consider regional anaesthesia (RA) to be a safer option for CS in this group.^1, 8^ The Royal College of Obstetricians and Gynaecologists guidelines recommend neuraxial anaesthesia as a safer technique as there is a lower risk of haemorrhage when compared to GA for CS in women with PP.^5^

Surveys done in the developed countries have shown variation in practice among anaesthesiologists regarding specific anaesthetic techniques in PP.^9-11^ There is a paucity of literature from the developing world regarding the characteristics and outcomes of PP cases undergoing CS in relation to specific anaesthesia mode. As a result, there is no standardized approach to anaesthetic management in PP, leaving the use of the technique at the discretion of individual anaesthesiologists in the absence of recommended guidelines.

This study aims to add to the limited literature on the role of different anaesthetic techniques for women undergoing CS with PP in the developing world. The aim of this study is to analyse the 14-year data from a tertiary care hospital regarding the anaesthetic management of parturients with PP to determine the best-suited mode of anaesthesia which can help in the development of guidelines and recommendations. It can be further utilized for the anaesthesia risk stratification, counselling, and delivery planning of women diagnosed with PP.

The objective of this retrospective study is to see an association between anaesthetic technique (neuraxial vs. general) with blood loss and maternal/neonatal outcomes in terms of mortality and/or admission to intensive care from 14-year cohort database.

## Methods

This retrospective study was conducted at Aga University Hospital, Karachi, Pakistan after approval from the Ethics Review Committee (ERC no: 4313-Ane-ERC-16). The hospital’s computerized database was used to identify cases of PP done during 14 calendar years inclusive of January 1, 2006, through December 31, 2019. The words “placenta previa” and “antepartum haemorrhage” as an indication of CS were searched in the hospital computer database. The medical record of these patients was then retrieved, and a predesigned data collection form was used to extract the data. For each patient, the following information was recorded: patients’ demographics, gestational age, parity, the nature of surgery (emergency or elective), the position of the placenta (from ultrasound report) grade of PP according to the relation of placenta to the cervical os (grade II defined as low lying or marginal, grade III as partially covering, and grade IV as completely covering the cervical os), the technique of anaesthesia (GA or RA), estimated blood loss grouped into the following categories: <500 mL, 501-1000 mL, 1001-1500 mL, and >1500 mL, intraoperative hypotension (systolic blood pressure <80% baseline), need of vasopressors, blood transfusion, presence of antenatal bleeding, and caesarean hysterectomy. The outcome of the mother and baby in terms of mortality and disposition either to the ward or intensive/high dependency was also assessed. The time of administration of anaesthesia was also recorded and allocated into 4 slots: 08:00 am-5:00 pm, 5:00 pm-8:00 pm, 8:00 pm-12:00 midnight, and 12:00 midnight–08:00 a m.

### Statistical Analysis

The collected data were entered and analysed using Statistical Package for the Social Sciences (SPSS) version 19.0 (IBM; Armonk, NY, USA). Mean and SD values were reported for quantitative variables and analysed by an independent sample *t*-test. The categorical observation was reported in terms of frequency and percentages and analysed by chi-square test. Estimating blood loss was grouped into the following categories: ≤500 mL, 501-1000 mL, 1001-1500 mL, and >1500 mL. The ordinal logistic regression model was used to observe the association with relevant factors and group estimated blood loss. The transfusion requirement was a binary outcome so binary logistic regression with a forward stepwise method was applied to observe the factors associated with transfusion. *P* ≤.05 was taken as indicating statistical significance.

## Results

Two hundred seventy-six consecutive cases of PP progressing to CS were identified during the study period. Around 36.24% (100/276) CSs were performed under RA and 63.76% (176/276) under GA. Use of RA was significantly less for when performed for emergency CS (*P* = .033) and for grade IV PP (*P* = .013) (Table 1).

**Figure 1.**
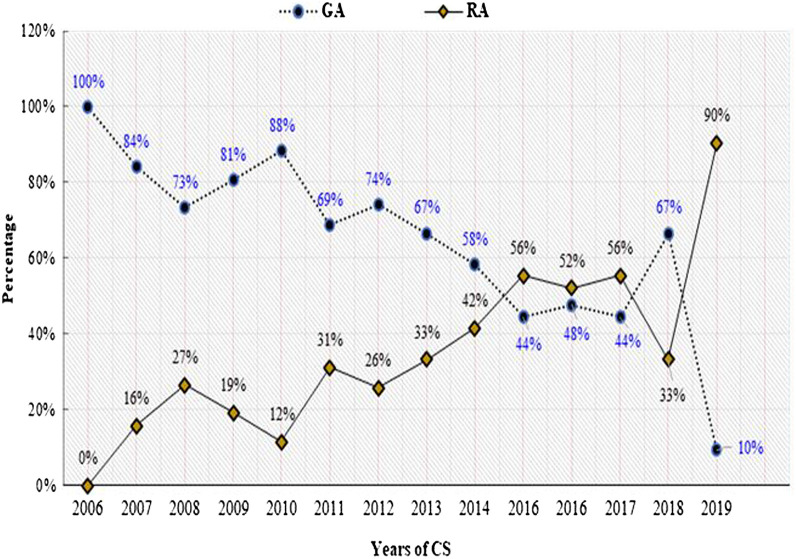
Comparison of technique of anaesthesia from the years 2006 to 2019.

Regarding the timing of surgeries, the maximum number of surgeries (n = 227, 82%) were done from 08:00 am to 5:00 pm, with RA being used in 36% of patients. There was no statistically significant difference in the use of RA when compared to other timings (5:00 pm-08:00 am). However, when the technique of anaesthesia was compared over 14 calendar years of the study period, a progressive rise in the trend of use of RA for CS in patients with PP was observed. Figure 1 shows the percentage use of GA or RA for patients undergoing CS from 2006 to 2019. It is observed that in 2006, GA was the technique of choice in 100% of patients, which progressively declined to 10% in 2019.

The use of different anaesthetic techniques for different grades of PP, undergoing emergency or elective CS, is shown in Table 2. Grades I and II PP were merged and used as 1 variable in the analyses. Two cases of emergency CS for grade II PP required conversion from RA to GA due to inadequate block. Overall, there were 171 (62%) patients with grade IV PP; GA was used as a technique of anaesthesia in 70% of these patients. There were altogether 6 patients who ended up in CS hysterectomy; all had grade IV PP and 2 were associated with the morbidly adherent placenta (MAP). All 6 patients requiring CS hysterectomy received GA as a technique of anaesthesia (Table 2).

In the comparison of factors associated with intraoperative blood loss, a statistically significant difference was found in the type of anaesthesia (*P* = .002), grade of PP (*P* = .014), and patients having CS hysterectomy compared to those not having CS hysterectomy (*P* = .0005) (Supplemental Table 1). Similarly, the factors responsible for the statistically significant requirement for blood transfusion included the type of anaesthesia (*P* = .0005), grade of PP (*P* = .005), and CS hysterectomy (*P* = .0005), with the addition of antenatal bleeding (*P* = .004), as most of these transfusions were done in the antenatal period (Supplemental Table 2).

When data were entered into relevant stepwise regression models, it was found that blood loss was significantly low with RA (*P* = .005) and posterior placenta (*P* = .042), while it was high in grade IV PP (*P* = .024) (Table 3). The odds of requiring blood transfusion were low in RA when GA was taken as reference [odds ratio (OR) = 0.122; 95% CI = 0.041-0.36, *P* = .0005) and posterior placenta (OR = 0.402; 95% CI = 0.201-0.804, *P* = .010), while they were high in grade IV (OR = 4.13; 95% CI = 0.90-19.80, *P* = .0681) (Table 4). Although patients with grade IV PP had 3.98 times more likely to have a transfusion, the difference was not significant. Among 171 (62%) patients with grade IV PP, 4 (2.33%) had MAP with estimated intraoperative blood loss of >1500 mL, requiring intraoperative blood transfusion and vasopressors.

On comparing maternal outcome with estimated blood loss, it was found that no maternal mortality was encountered among study subjects; however, a statically significant association was observed for maternal disposition (*P* = .009). Among patients having an estimated blood loss of >1500 mL, 7.2% went to the ward, 25% went to the high-dependency unit (HDU), and 50% went to the intensive care unit (ICU) (Supplemental Table 3). Regarding intraoperative events, hypotension was recorded in 19 (10.7%) patients, 18 of whom received GA. A statistically significant association was observed between estimated blood loss and incidence of hypotension (0.0005) as 63% had a blood loss of >1500 mL, requiring treatment with vasopressors.

As for neonatal outcomes with estimated blood loss, no statistically significant association was observed between neonatal mortality (*P* = .075) and disposition to neonatal intensive care unit (NICU) (*P* = .734) (Supplemental Table 3). Records revealed a total of 28 (10%) neonatal deaths, out of which 21 were born to mothers receiving GA. Only in 2 cases of neonatal deaths, mothers had a blood loss of >1100 mL, and both received GA. Of the 36 neonates requiring NICU admission, GA was used as the anaesthetic technique for CS in 26 cases. There were only 6 cases of NICU admission where the mother had blood loss of more than 1100 mL with GA as the technique of choice.

## Discussion

In this retrospective analysis of 276 women with PP, there are 3 important findings. First, RA was associated with less blood loss and better maternal and neonatal outcomes compared to GA. Second, the technique of anaesthesia used in most cases (63.76%) was GA, with statistically significant higher use among patients operated for emergency CS and those having grade IV PP. Third, a major turn in the trend of anaesthetic technique was observed for patients undergoing CS for PP; a decline in the use of GA was observed from 100% use in 2006 to only 10% utilization of GA in 2019.

In our study, we found no significant permanent morbidity or mortality attributed to either anaesthetic technique. However, results from our study suggested that RA is associated with less blood loss and transfusion requirement, which is in congruence with the findings of the previous literature on this subject.^12-14^ The major argument that could be put against the benefits of RA found in our study and the previous literature is the difference in the risk factors of patients between the 2 groups. The choice of anaesthesia in our study was based mostly on the urgency of surgery and the grade of PP, resulting in higher use of GA in patients presenting with grade IV PP or in an emergency. These factors have been shown to aggravate the risk of bleeding and transfusion.^15-18^ As the groups receiving RA and GA in our retrospective analysis differed in prior risk factors, we applied regression statistics in our data to control for many interacting factors affecting blood loss. This process clearly indicated that compared to GA, RA was associated with significantly lower estimated blood loss and lower transfusion requirements.

Considering the maternal and neonatal outcomes, our data showed no maternal mortality; however, 4 patients required ICU admissions and 8 required admissions to HDU. Although GA was the choice of anaesthesia in the majority, the main reason for ICU/HDU admission was blood loss secondary to grade IV PP. Previous studies have shown that although blood loss was heavier among women with major PP (grade II and IV), the rate of other adverse outcomes and postpartum complications were similar among different grades of PP.^19^

In our retrospective analysis, 28 neonatal deaths were reported; however, no association was observed between neonatal deaths and blood loss. Out of 28 deaths, 21 (75%) cases have GA as the technique of anaesthesia for mothers. Additionally, out of 36 NICU admissions, 26 (72%) were delivered by CS under GA. Neonatal outcomes might be hampered following GA due to transplacental passage of anaesthetics drugs or cases of maternal hypotension. The previous study has also shown a higher rate of neonatal asphyxia and admission to NICU for babies delivered under GA.^13^ In another study, GA was found to be an independent risk factor for adverse neonatal outcome.^20^

Mostly, the reason for advocating GA for PP is the fear of sympathectomy induced by RA causing hypotension, further aggravating the low arterial pressure caused by haemorrhage. In our retrospective analysis, we observed that 95% of the patients having episodes of hypotension received GA as the technique of anaesthesia. Therefore, the probable reason for hypotension was related to blood loss and not the technique of anaesthesia. Previous literature has shown that the use of light GA during haemorrhagic episodes causes intense vasoconstriction, leading to decreased organ perfusion and oxygenation predisposing the mother to major organ failure in the postoperative period, whereas RA has shown to provide a protective effect during physiological responses of the body to haemorrhage.^21,22^ Animal studies have shown slower heart rates, greater cardiac stroke volume, higher arterial pH and bicarbonate concentration, and lower catecholamine and lactate concentration, in addition to an increase in uterine vascular resistance in RA as compared to GA controls during haemorrhagic episodes.^21,22^

In our study, we observed all patients with the MAP had grade IV PP and 50% of them ended up in CS hysterectomy. This finding is consistent with previous studies showing a significant association of grade IV PP with MAP (OR 3.2) and hysterectomy (5.1).^6,23^ However, a recent study^19^ showed that MAP was similar among major and minor PP, and no difference was observed in the rate of hysterectomy.

None of the patients in our study required conversion from RA to GA due to excessive bleeding or for the reason of proceeding to caesarean hysterectomy. In our study, all the cases proceeding to caesarean hysterectomy were given GA from the start which is the recommendation from the older literature.^24^ However, Chestnut et al^25^ recommend that elective caesarean hysterectomy should not be a contraindication for neuraxial technique. One case report mentioned the successful use of single-shot spinal anaesthesia without conversion to GA in patients undergoing CS proceeding to hysterectomy.^26^ One of the recent studies has reported the 21% conversion rates of intraoperative conversion of RA to GA because of the need to perform a hysterectomy for patients undergoing CS for PP with suspected MAP.^27^ However, they encountered 3 difficult intubations, highlighting the controversy surrounding the unsecured airway during CS with increased risk of massive haemorrhage. The investigators of this study recommend that in resource-limiting setting, it is better to secure the maternal airway in patients with anticipated surgical complexity or a difficult airway. In addition, the other reason for choosing GA in patients with anticipated caesarean hysterectomy is to avoid giving GA in the setting of RA-induced sympathectomy, which may lead to severe maternal hypotension. One case report mentioned facing haemodynamic instability when combined spinal–epidural anaesthesia was used for CS proceeding to hysterectomy in a placenta accreta case.^28^ Other reasons for the need for GA conversion could increase the duration and surgical pain in patients requiring caesarean hysterectomy requiring intravenous supplemental sedation and analgesia in amounts that could approach typical doses used for GA, albeit without definitive airway management.

The study is limited by the fact that patient data was collected from a single tertiary care centre, therefore, applicability of the results to other settings maybe limited. In addition, due to the retrospective nature of this study, completeness was limited by the documentation available in the medical records. The analysis of pre- and postoperative haemoglobin values was complicated by the fact that women received a blood transfusion at various times, before, during, and after surgery, and from retrospective analysis, it was not possible to ascertain when the blood samples were obtained in relation to blood transfusion. Therefore, we were unable to analyse the data on pre- and postoperative haemoglobin. Another limitation was the inability to record anaesthesia to the delivery time interval between the RA and GA groups of patients which could have given us a better picture of low neonatal outcomes delivered under GA. The strength of this study is its analysis of 14 years of anaesthetic management of PP from a developing country, which adds to the growing literature mostly from the developed nation.^14^

Due to the presence of confounding variables in terms of the difference in the baseline risk factors, an inherent limitation of retrospective data collection, and the paucity of good prospective trials, it is difficult to find objective evidence on which to base the arguments for or against GA or RA for PP. However, this retrospective analysis of 14 years for anaesthetic management of patients presenting for CS for PP finds data supporting the use of RA in terms of reduced blood loss, need for transfusion, and better maternal and neonatal outcomes.

## Conclusion

In conclusion, PP is one common cause of massive obstetric haemorrhage associated with transfusion requirement and worst maternal and neonatal outcomes. Both GA and RA options have been utilized; however, there is a trend towards the use of RA due to its suggested benefits of less blood loss, need for transfusion, and better maternal and neonatal outcomes.

## Figures and Tables

**Table 1.** Characteristics of Parturient According to Anaesthesia Technique (n = 276)

Variables	Anaesthetic Technique		*P*
	GA (n = 176)	RA (n = 100)	
Age (years)	30.66 ± 4.84	29.54 ± 4.32	.056
Weight (kg)	70.74 ± 12.18	69.68 ± 9.98	.463
Height (cm)	156.92 ± 6.52	156.50 ± 7.20	.621
BMI (kg m^–1^)	28.78 ± 5.25	28.39 ± 4.13	.525
Gestational age (weeks)	36.93 ± 1.70	36.93 ± 1.70	.287
Parity			.151
0	57 (32.4%)	33 (33%)	
1	74 (42%)	51 (51%)	
2-6	45 (25.6%)	16 (16%)	
Nature of surgery			.033
Emergency	68 (38.6%)	26 (26%)	
Elective	108 (61.4%)	74 (74%)	
Antenatal bleeding	45 (25.6%)	17 (17%)	.101
Grade of placenta previa			.013
Grade I	0 (0%)	1 (1%)	
Grade II	16 (9.1%)	15 (15%)	
Grade III	39 (22.2%)	34 (34%)	
Grade IV	121 (68.8%)	50 (50%)	

BMI, body mass index; GA, general anaesthesia; RA, regional anaesthesia.

**Table 2.** The Grade of Placenta Previa, the Nature of Surgery, and Anaesthesia Technique

Anaesthesia Technique	Grade I and II		Grade III		Grade IV		Total
	EM	EL	EM	EL	EM	EL	n = 276
GA	8^*^ (61.5%)	8 (42.1%)	14^*^ (73.7%)	25 (46.3%	46^**^ (74.2%)	75^***^ (68.8%)	176 (63.7%)
RA	5 (38.5%)	11 (57.9%)	5 (26.3%)	29 (53.7%)	16 (25.8%)	34 (31.2%)	100 (36.3%)

^*^Includes 2 cases where spinal anaesthesia was performed but failed to achieve a complete block and were therefore converted to GA.

^**^Includes 2 cases proceeding to hysterectomy.

^***^Includes 4 cases with morbidly adherent placenta proceeding to hysterectomy.

EL, elective; EM, emergency; GA, general anaesthesia; RA, regional anaesthesia.

**Table 3.** Ordinal Logistic Regression Model, Showing Predictors Associated with Estimated Blood Loss (n = 276)

Parameter	Regression Coefficient	Standard Error (SE)	*P*
*Technique of anaesthesia*			
Regional	–0.703	0.25	.005
General	Ref*		
*Position of placenta previa*			
Posterior	–0.486	0.24	.042
Anterior	Ref		
*Grade of placenta previa*			
Grade I and II	Ref		
Grade III	0.18	0.40	.661
Grade IV	0.84	0.37	.024

Nagelkerke *R* ^2^ = 0.117. Model accuracy summary: Akaike’s information criterion = 438.23. Bayesian information criterion = 467.19 and log likelihood = –211.12. *Ref: GA taken as reference.

**Table 4.** Binary Logistic Regression Model, Showing Predictors Associated with Transfusion (n = 276)

Parameter	OR	95% CI	*P*
*Technique of anaesthesia*			
Regional	0.122	0.041-0.36	.0005
General	Ref		
*Position of placenta previa*			
Posterior	0.402	0.201-0.804	.010
Anterior	Ref		
*Grade of placenta previa*			
Grade I and II	Ref		
Grade III	1.38	0.25-7.59	.713
Grade IV	4.13	0.90-19.80	.0681

Model accuracy = 83.1%, Nagelkerke *R* ^2^ = 0.21. Forward stepwise method (likelihood ratio) was used. Age corresponds to gestational age, and BMI and parity were excluded from the model.

OR, odds ratio.
